# Characterization of the complete chloroplast genome of *Gastrochilus sinensis* (Orchidaceae, Epidendroideae), a beautiful epiphytic orchid from China

**DOI:** 10.1080/23802359.2023.2301033

**Published:** 2024-01-11

**Authors:** Junyi Zhang, Min Liao, Bo Xu, Hai He

**Affiliations:** a College of Life Sciences, Chongqing Normal University, Chongqing, China; b CAS Key Laboratory of Mountain Ecological Restoration and Bioresource Utilization & Ecological Restoration and Biodiversity Conservation Key Laboratory of Sichuan Province, Chengdu Institute of Biology, Chinese Academy of Sciences, Chengdu, China; c College of Life Sciences, University of Chinese Academy of Sciences, Beijing, China

**Keywords:** *Gastrochilus sinensis*, chloroplast genome, phylogeny

## Abstract

*Gastrochilus sinensis* is a beautiful epiphytic orchid with high ornamental value. In this study, the first complete chloroplast genome sequence of *G. sinensis* was determined using next-generation sequencing (NGS). The *de novo* assembled chloroplast genome was 148,020 bp in length, including a pair of inverted repeat regions (IRs; 25,987 bp), a small single-copy region (SSC; 11,045 bp), and a large single-copy region (LSC; 85,001 bp). The chloroplast genome encodes 109 unique genes, including 75 protein-coding genes (PCGs), 30 tRNA genes, and four rRNA genes. The total GC content of the chloroplast genome was 36.8%. The phylogenetic analysis showed a close relationship between *G. sinensis* and *G. formosanus* species. The complete chloroplast genome provides fundamental information for genetic diversity and phylogenetic relationships in *Gastrochilus*.

## Introduction

*Gastrochilus sinensis* Z.H. Tsi 1996, is a beautiful epiphytic orchid and distributed in southern China (Tsi 1996; Chen et al. 2009; Cheng et al. 2022). It has a showy labellum, which is divided into a recurved epichile and a saccate hypochile, with high ornamental value (Pridgeon et al. 2014; Liao et al. 2022). With rapid development of the second-generation sequencing technology, the chloroplast genome information was widely used for studying taxonomy, phylogeny, evolution, conservation, and ecology in plants (Jheng et al. 2012; Zhang, Liao, et al. 2022). Until now, only 10 plastid genomes of *Gastrochilus* have been reported (Liu et al. 2020), which has greatly hindered the aforementioned studies in this genus. Therefore, we reported the first complete chloroplast genome of *G. sinensis* based on the whole-genome Illumina sequencing dataset in this study.

## Materials and methods

**Figure 1.**
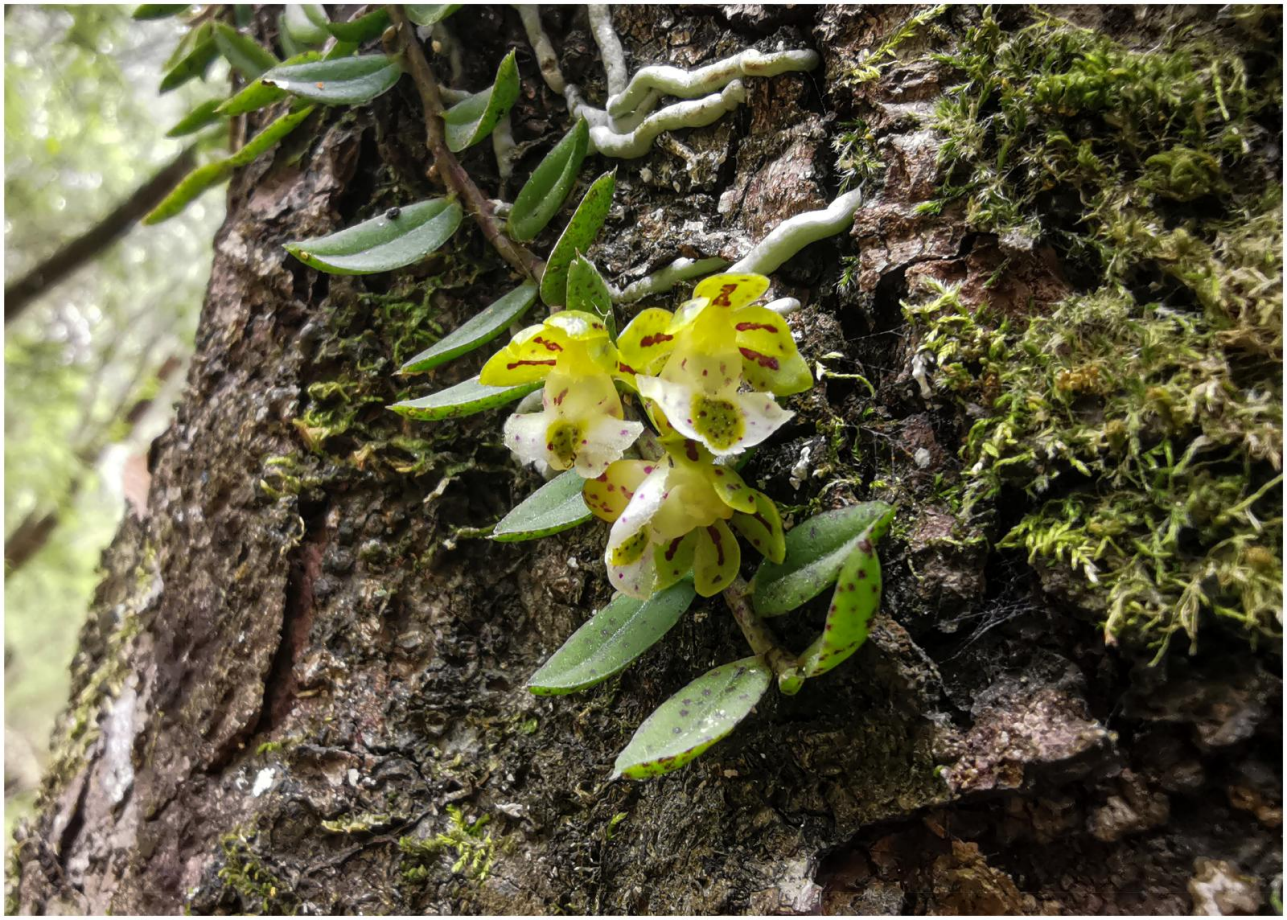
Flowering plant image of *Gastrochilus sinensis* (this unpublished photo, taken in Sanjiang, Wenchuan County, Sichuan Province, China by Mr. Yuehong Cheng, is used with permission).

Fresh leaves of *Gastrochilus sinensis* (Figure 1; voucher *ZJY181*; contact person: Bo Xu, xubo@cib.ac.cn) were collected from Sanjiang (31°7′14.95″N, 103°13′25.60″E), Wenchuan County, Sichuan Province, China, and a voucher specimen was deposited at the Herbarium of Chengdu Institute of Biology (CDBI). Total genomic DNA was extracted from silica-gel dried leaves through Plant DNA Isolation Kit (Cat. No. DE-06111, Foregene, Chengdu, China). Next-generation sequencing (NGS) was performed via Illumina paired-end technology. *De novo* assembly of the chloroplast genome was carried out using GetOrganelle v1.7.2 (Jin et al. 2020). The average read mapping depths of the assembled genome were 2729× (Figure S1). The assembled chloroplast genome was annotated using PGA (Qu et al. 2019) and manually corrected for the start and stop codons. The final genome map of *G. sinensis* was generated using CPGview (http://www.1kmpg.cn/cpgview). The annotated chloroplast genome was submitted to the GenBank under the accession number OQ784257.

The phylogenetic tree was constructed based on 75 protein-coding genes (PCGs) shared by 20 complete chloroplast genomes of the Aeridinae, with *Thrixspermum japonicum* (Miq.) Rchb. f. 1878, as outgroup species. Sequences were aligned via MAFFT v7.475 (Katoh and Standley 2013). The nucleotide substitution model for the matric was estimated using the software jModelTest v.2.1.6 (Posada 2008) and the best fit model (TVM + F + R3) was selected using the corrected Akaike information criterion (AIC). A maximum-likelihood (ML) method for phylogenetic analysis was performed via IQ-Tree v1.6.10 (Nguyen et al. 2015) and visualized in FigTree v1.4.4 (http://tree.bio.ed.ac.uk/software/figtree).

## Results

**Figure 2.**
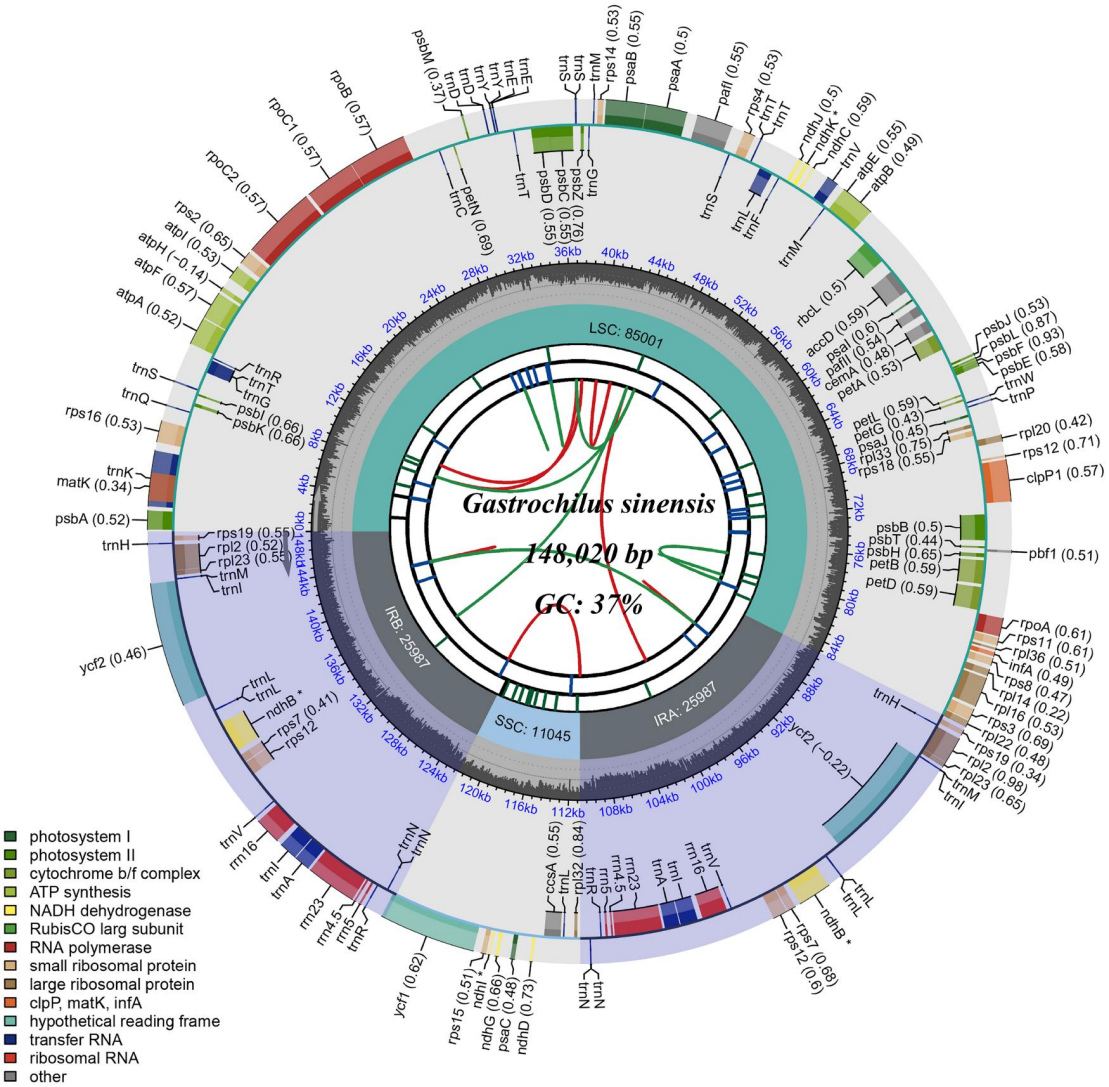
The circle map of complete chloroplast genome of *Gastrochilus sinensis*. The map was generated by CPGView. Genes located on the inner and outer of circle are transcribed clockwise and anticlockwise, respectively. The dark grey inner circle indicates GC content. Large single-copy (LSC), small single-copy (SSC), and inverted repeats (IRA and IRB) are indicated in the inner layer. The functional classification of the genes is provided in the bottom left corner.

**Figure 3.**
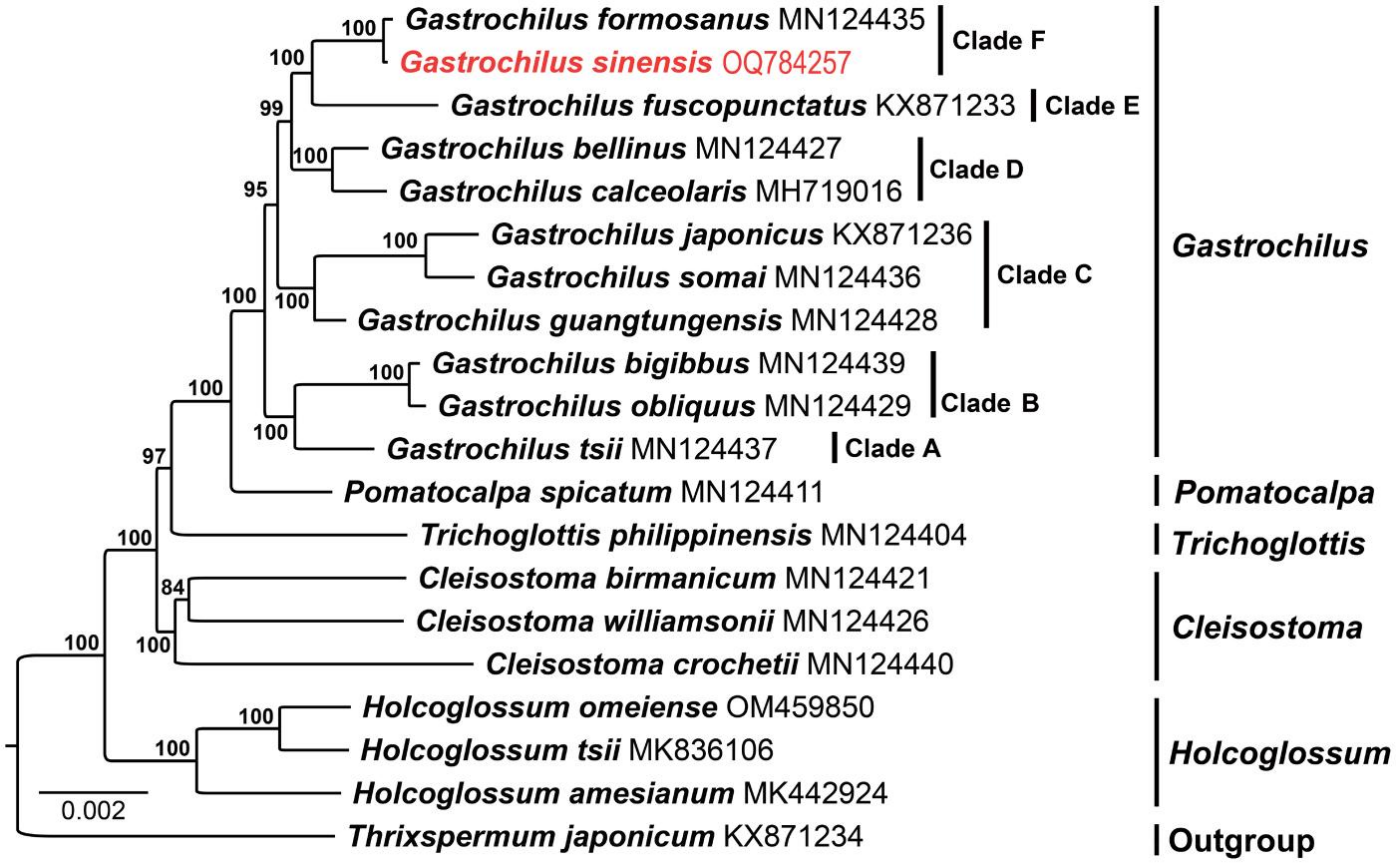
The ML phylogenetic tree for *Gastrochilus sinensis* based on 20 chloroplast genomes in Aeridinae. The accession numbers of used sequences follow the species names, and the newly sequenced genome is shown in red font.

The complete chloroplast genome of *Gastrochilus sinensis* is 148,020 bp in length, containing a quadripartite structure that consists of a large single-copy (LSC) region of 85,001 bp and a small single-copy (SSC) region of 11,045 bp with two inverted repeat (IR) regions of 25,987 bp (Figure 2). The overall GC content was 36.8%, which is higher than either LSC regions (34.0%) or SSC (28.5%) region, but lower than the IR (43.1%) region. It encodes 109 unique genes, including 75 PCGs, 30 tRNAs, and four rRNAs. Introns were detected in 20 genes, where 18 genes (*atpF*, *ndhB*, *petB*, *petD*, *rpl16*, *rpl2*, *rpoC1*, *rps12*, *rps16*, *trnA-UGC*, *trnE-UUC*, *trnG-UCC*, *trnH-GUG*, *trnI-GAU*, *trnK-UUU*, *trnL-UAA*, *trnT-CGU*, and *trnV-UAC*) had a single intron, and two genes (*clpP1* and *pafI*) had two introns (Figure S2). The trans-splicing gene *rps12* had three unique exons (Figure S3). The phylogeny reconstructed based on 75 PCGs shared by 20 species in the subtribe Aeridinae strongly supports the fact that 11 species of *Gastrochilus* formed a monophyletic group, and *G. sinensis* is closely clustered with *G. formosanus* (Hayata) Hayata 1917 (Figure 3).

## Discussion

The genome size, gene content, and order are not significantly different from other published chloroplast genomes in *Gastrochilus* (Liu et al. 2020). The inferred phylogenetic tree analysis also confirmed the systematic position of *G. sinensis* supported by data on filtered plastid and nuclear loci (Zhang, Cheng, et al. 2022), that *G. sinensis* is a member of the monophyletic genus *Gastrochilus* in the subtribe Aeridinae of the tribe Vandeae of Orchidaceae. The other species of this genus have been clearly distinguished, but the topology of the inferred phylogeny is different from those of recently published phylogenies (Liu et al. 2019), such as the systematic positions of the clade C and D are opposite to Liu et al. (2019) and Zhang et al. (2023), suggesting that further research on phylogenetic relationships in *Gastrochilus* is necessary. The research results will be used for authenticating the plant materials of *G. sinensis* and for analyzing the genetic diversity and phylogenetic relationships in *Gastrochilus*.

## Supplementary Material

Supplemental Material

## Data Availability

The data that support the findings of this study are openly available in GenBank number OQ784257 (https://www.ncbi.nlm.nih.gov/nuccore/OQ784257) and the related BioProject, raw sequencing files in SRA, and the Bio-Sample number are PRJNA953444, SRR24108513, and SAMN34116696, respectively.
