# Supplementary material for: Characterization of the complete chloroplast genome of Gastrochilus sinensis (Orchidaceae, Epidendroideae), a beautiful epiphytic orchid from China
Source: Mitochondrial DNA B Resour. 2024 Jan 11;9(1):100–3. doi: 10.1080/23802359.2023.2301033 (PMC10791082; doi:10.1080/23802359.2023.2301033)
Supplement: Supplemental Material [file TMDN_A_2301033_SM6015.docx]

**Figure S1.** The read coverage depth map of the assembled genome.

**Figure S2.** Schematic maps of cis-splicing genes in the chloroplast genome of *Gastrochilus sinensis*. Maps generated using CPGView. The gene names are shown on the left, and the gene structures are on the right.

**Figure S3.** Schematic maps of the trans-spliced gene *rps12*. Maps generated using CPGView.

**Figure S1.**

**
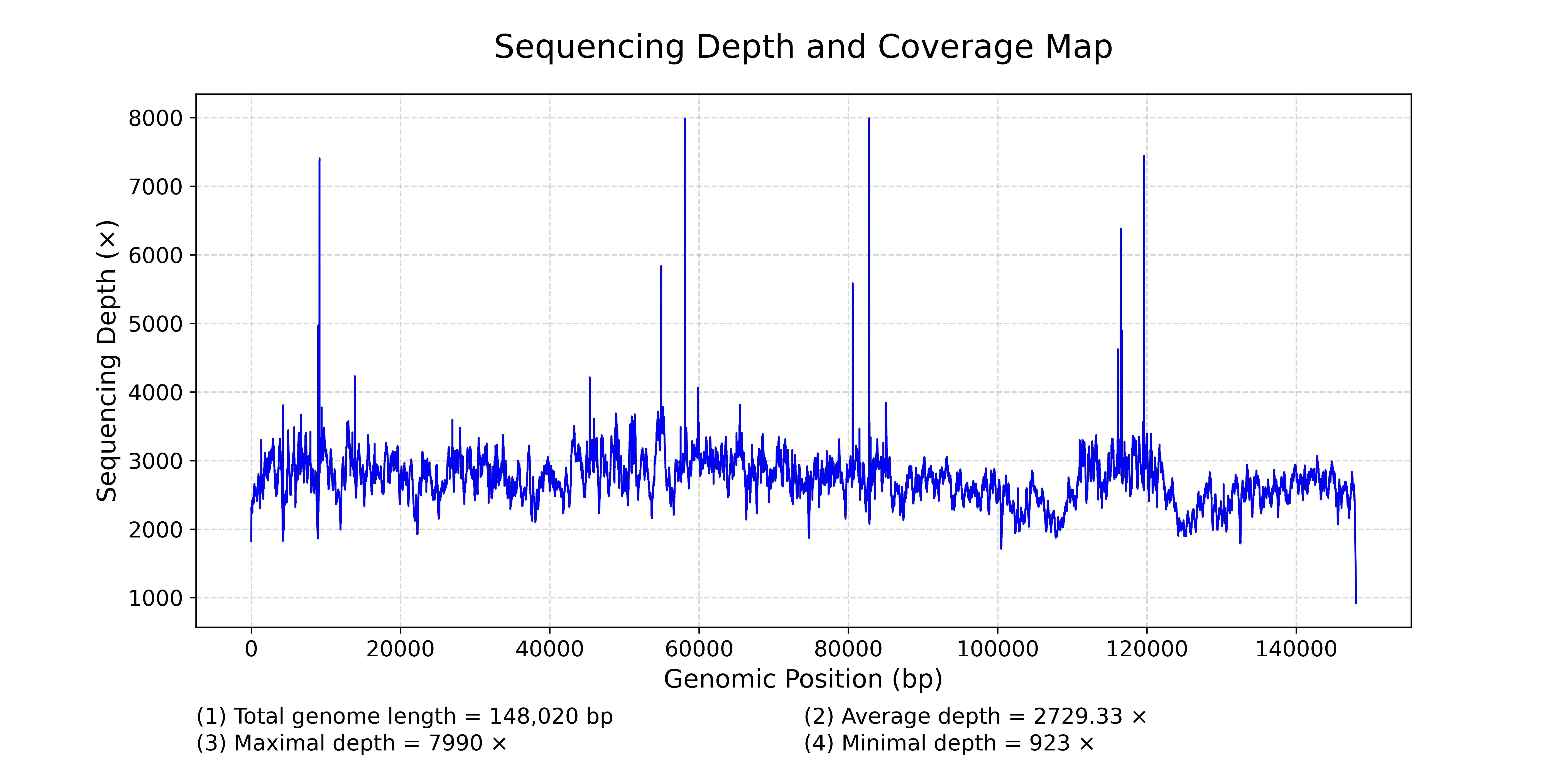
**

**Figure S2.**

**
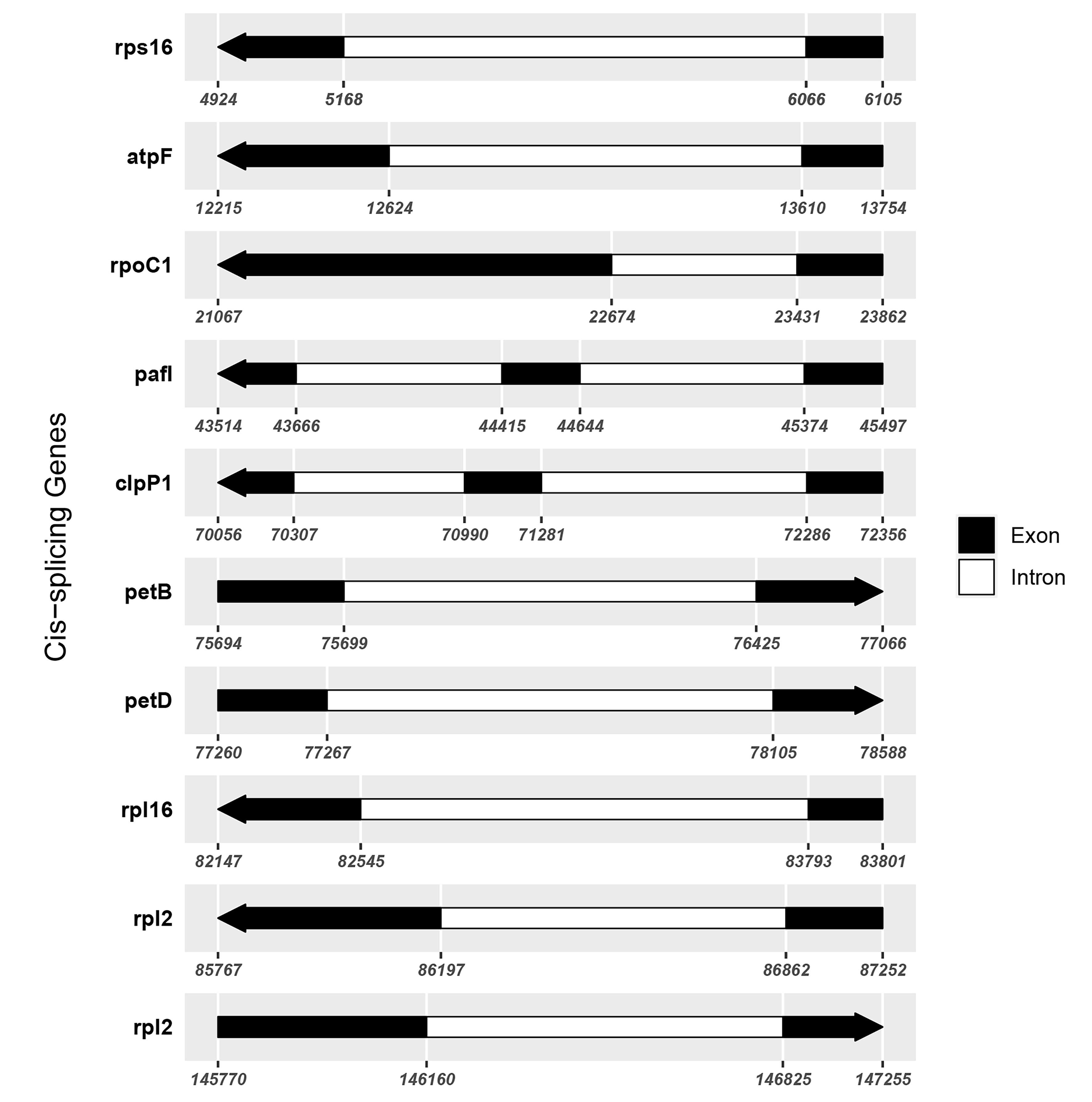
**

**Figure S3.**

**
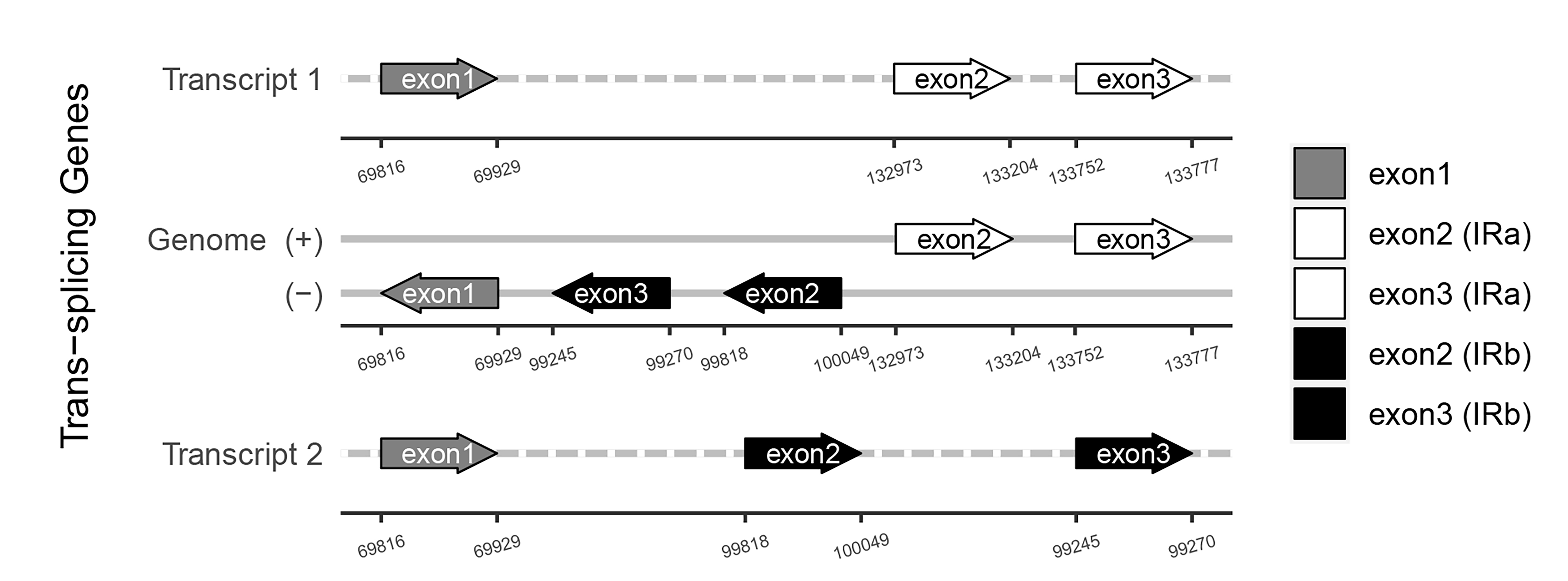
**
